# Annotations

**Published:** 1895-10-26

**Authors:** 


### Annotations.

### Spurious Hospitals.

Attention is drawn in *Truth* to the fact that, after some debate, the Fulham Vestry has allowed the use of their vestry hall for a meeting in aid of the Jubilee Hospital. The report published a few weeks back in The Hospital, in which the institution was entirely condemned on medical and sanitary grounds, is referred to; and it is pointed out that if the Jubilee Hospital is not actually within the Fulham sanitary area, it is near enough to be a matter of anxiety to Fulham residents, and that the vestry would be better occupied in setting their own or the Kensington medical officer to work to abate the nuisance, than in giving their patronage assistance for the purpose of perpetuating it. In the debate it appears to have been stated that the institution had been doing much good work among the poor of the neighbourhood, and no doubt this statement had considerable weight. It must be remembered, however, such a statement could only have been made by people who either were entirely ignorant of what they were talking about, or were willing to accept a very low standard of what is good enough for the poor. The scandal lies in calling the place a hospital; if they will call it a "refuge" or a "shelter" no one will complain, for no patients will then go, and the abuse will die a natural death. But to induce patients, by the advertised name of "hospital," to enter what is not a hospital, is not only a wrong to those who are so misled, but is an insult to the numerous charities which have a right to that honoured name.

### The Harveian Oration: Then and Now.

Dr. W. S. Church, the Harveian orator, told the Fellows of the College of Physicians last week two things, which, if both true, serve well to show the difference in spirit and feeling between the doctors of Harvey's day and the medical profession of our own. On the one hand, "Harvey's physiological researches had little or no immediate effect on medicine"; on the other, "the brilliancy of modern discoveries and the vast increase of our knowledge may for a time react prejudicially on medicine." In other words, Harvey provided his medical contemporaries with a scientific lantern which they declined to use; whereas in our time everybody is so busy staring at the stars and comets of new discovery which are so constantly being brought within scientific vision that many of us cannot practise homely, everyday physic well because we are "blinded with excess of light." It is much to be feared that Dr. Church is right in both particulars. If a jury of present-day medical men were to sit in judgment upon Harvey's contemporaries, it is quite certain that a verdict of uncommon, not to say wicked, stupidity would promptly be pronounced upon these ancient gentlemen. But perhaps, if they in turn could for a brief period recover the use of tongue and brain, they might retort that the person who admires the light and takes the lantern in his hand, but has not intelligence enough to improve his practice by it, is just as stupid as he who insists upon ignoring the light altogether. There is clearly a contest between the ancients and some of the moderns, not altogether to the advantage of the latter. Dr. Church very ingeniously called in one who was both an ancient and a modern to restore the balance. Sydenham, as everybody will admit, even those who know nothing about him but his name, was both a philosopher and a man of science in medicine. On this question of science and practice Sydenham wrote thus: "The art of medicine can be properly learned only from its practice and exercise." That verdict Dr. Church, and every other Fellow of the College of Physicians of any mind, definitely endorsed. It cannot be too often repeated that medicine is an art, and that though science is one of the very best guides in the world, the art which is to benefit the patient can only be learnt by steady, watchful, and life-long practice at the bedside.

### An Artificial Larynx.

A case reported from Melbourne in which Professor Anderson Stuart is stated to have provided a patient with an artificial larynx, has led many papers to deal with this subject as if this was an entirely novel invention. The fact is, however, that more than twenty years ago Professor Billroth applied an apparatus of the sort, devised by Dr. Gussenbauer, to the throat of a patient for whom he had excised the larynx. Many modifications of the instrument have since been introduced, but in principle it remains very like it was at first. In all operations for the removal of the larynx the first step is to make an opening into the trachea below the disease, and to insert a tube through which breathing can be carried on, and which, by accurately plugging the air tube prevents blood and other matters gaining access to the lungs. The result, then, of the operation is to leave a three-way opening, one way leading down the trachea, another out into the air in front of the neck, and one into the throat cavity. As in an ordinary case of tracheotomy, respiration takes place through the tube, and by closing the orifice of the latter air can be expelled through the mouth. In tracheotomy, when this is done, the air is driven over the vocal cords and sound is produced, but where the vocal cords have been destroyed, or even, as in some cases, the whole larynx has been removed, no such effect is produced. If, however, the upper opening in the tube be continued upwards, in the form of a branch passing towards the mouth, it is possible to place a vibrating reed like that of a harmonium within this latter portion in such a manner as to produce a sound when air is made to blow over it. This is the artificial larynx. We have, then, a three-way tube, one branch which is always open, going towards the lung, another through which air is drawn in, opening in front of the neck, and the third, through which the air is driven out, and in which is placed the reed, passing up towards the mouth. These are the essentials. It is obvious, however, that a three-way tube like this is a large affair, and must be taken to pieces for introduction and extraction, and that in regard to this and the changing of the reeds, and the arrangement of valves by which the air may be made to take the right direction, there is considerable play for ingenuity, and it is in reference to these points that most of the modifications and variations in the artificial larynx have been made.

